# Echo-Imaging Exploits an Environmental High-Pass Filter to Access Spatial Information with a Non-Spatial Sensor

**DOI:** 10.1016/j.isci.2019.03.029

**Published:** 2019-04-18

**Authors:** A. Leonie Baier, Lutz Wiegrebe, Holger R. Goerlitz

**Affiliations:** 1 Department Biology II, Ludwig Maximilians University Munich, 82152 Martinsried, Germany; 2 Acoustic and Functional Ecology Group, Max Planck Institute for Ornithology, 82319 Seewiesen, Germany

**Keywords:** Acoustics, Bioacoustics, Biological Sciences, Zoology

## Abstract

Echo-imaging evolved as the main remote sense under lightless conditions. It is most precise in the third dimension (depth) rather than in the visually dominating dimensions of azimuth and elevation. We asked how the auditory system accesses spatial information in the dimensions of azimuth and elevation with a sensory apparatus that is fundamentally different from vision. We quantified echo-acoustic parameters of surface-wave patterns with impulse-response recordings and quantified bats' perceptual sensitivity to such patterns with formal psychophysics. We demonstrate that the spectro-temporal auditory representation of a wave pattern implicitly encodes its spatial frequency. We further show that bats are much more sensitive to wave patterns of high spatial frequencies than of low spatial frequencies. We conclude that echo-imaging accesses spatial information by exploiting an inherent environmental high-pass filter for spatial frequency. The functional similarities yet mechanistic differences between visual and auditory system signify convergent evolution of spatial-information processing.

## Highlights

• Echo-imaging displays the depth dimension, but elevation or azimuth must be computed
• Yet, the auditory representation of a surface pattern encodes its spatial frequency
• Echolocating bats spot high spatial frequencies much better than low ones
• Vision and hearing share functional similarities yet show mechanistic differences

## Graphical Abstract

**Figure:**
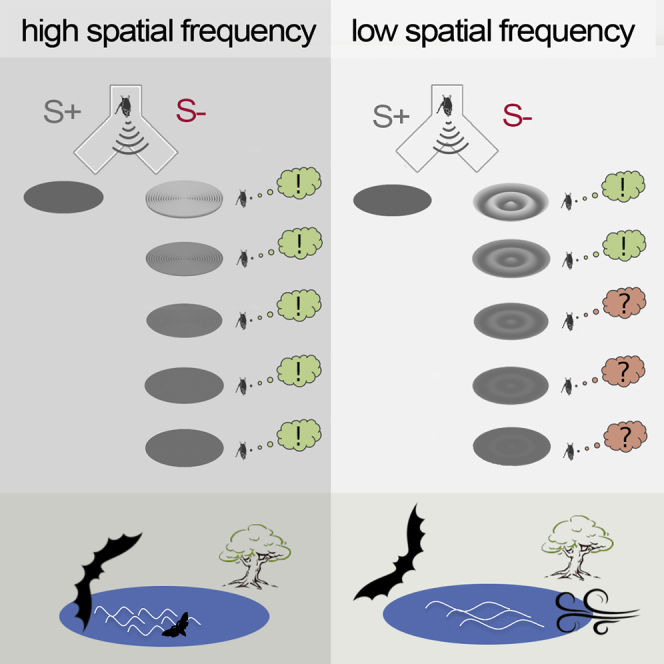

## Introduction

Echo-imaging evolved as the main sense for orientation and foraging under lightless conditions. Bats, one of the most notable taxa using echo-imaging, fly and forage in complex 3D environments. In the 60 years since Griffin's seminal *Listening in the Dark* (Griffin, 1958) the number of published studies on echo-imaging has grown exponentially, and yet, we do not fully understand how the auditory system solves the same task as the visual system with a fundamentally different sensory apparatus. The eye's retina possesses a two-dimensional anatomy, which supports high spatial acuity and resolution along the two dimensions of azimuth and elevation. Perception of the third dimension, depth, arises from computations in the visual cortex comparing the images of the left and right eye. In contrast, echo-imaging supports high acuity in depth perception but is poorly set up for displaying azimuth and elevation: the time delay between sound emission and echo return explicitly encodes target range, i.e., depth (Simmons, 1971, Simmons, 1973), but all incoming sounds—from all directions—are superimposed at the eardrums. Any spatial information is lost in the process and must later be neurally computed, including the most basic information such as the direction of a single sound source (Rayleigh, 1907). In vision, azimuth and elevation possess a topographic representation in the visual cortex (Zeki, 1978). In echo-imaging of bats, target range possesses a topographic representation in the auditory cortex (Hagemann et al., 2010, Hoffmann et al., 2008, O'Neill and Suga, 1979).

To detect, localize, and identify objects of interest (e.g., prey), bats need to analyze echoes reflected off target objects amid echoes reflected off non-target structures, the so-called clutter. When prey and clutter are close by in azimuth and elevation and/or when their distance on the depth axis falls below bats' threshold for target range differences of approximately 1 cm (Simmons, 1973), one would expect the prey detection task to become impossible. Yet, many bat species occupy foraging niches where they detect prey directly on surfaces: active-gleaning bats take prey directly off leaves, despite a potentially large number of clutter echoes (Geipel et al., 2013), and trawling bats take prey off water surfaces (Kalko et al., 1998, Ruedi and Mayer, 2001, Weinbeer et al., 2006), despite water turbulences potentially introducing clutter echoes as well.

Water turbulence can be described in terms of temporal and spatial frequencies. Temporal frequency quantifies the change of wave amplitude at one point in space as a function of time. Spatial frequency quantifies the change of wave amplitude at one point in time as a function of space. Wind generates high-amplitude waves of low spatial and temporal frequencies (Bleckmann and Rovner, 1984), whereas moving prey items generate low-amplitude waves of high spatial and temporal frequencies (Bleckmann, 1985). Many predatory species across the animal kingdom can discriminate between different wave sources by analyzing both spatial and temporal frequencies and amplitudes of the waves (Bleckmann, 1985).

Recently, we have shown that echo-imaging has access to temporal frequency (Baier et al., 2018, Baier and Wiegrebe, 2018). As echo-imaging cannot map spatial features in the explicit way the visual system does, we hypothesized that environmental spatial features are mediated by common cues present in echoes. We test this hypothesis pursuing two main objectives: (1) quantify the echo-acoustic properties of different wave patterns and (2) quantify the sensitivity of echo-imaging in detecting them. We found that the echo-acoustic properties of wave patterns vary systematically with spatial frequency and that the echolocating bat *Phyllostomus discolor* is much more sensitive to wave patterns of high spatial frequencies than of low spatial frequencies. We conclude that echo-imaging accesses spatial information, which the visual system accesses by means of a perceptual high-pass filter, by exploiting a pre-existing environmental high-pass filter.

## Results

**Figure 1:**
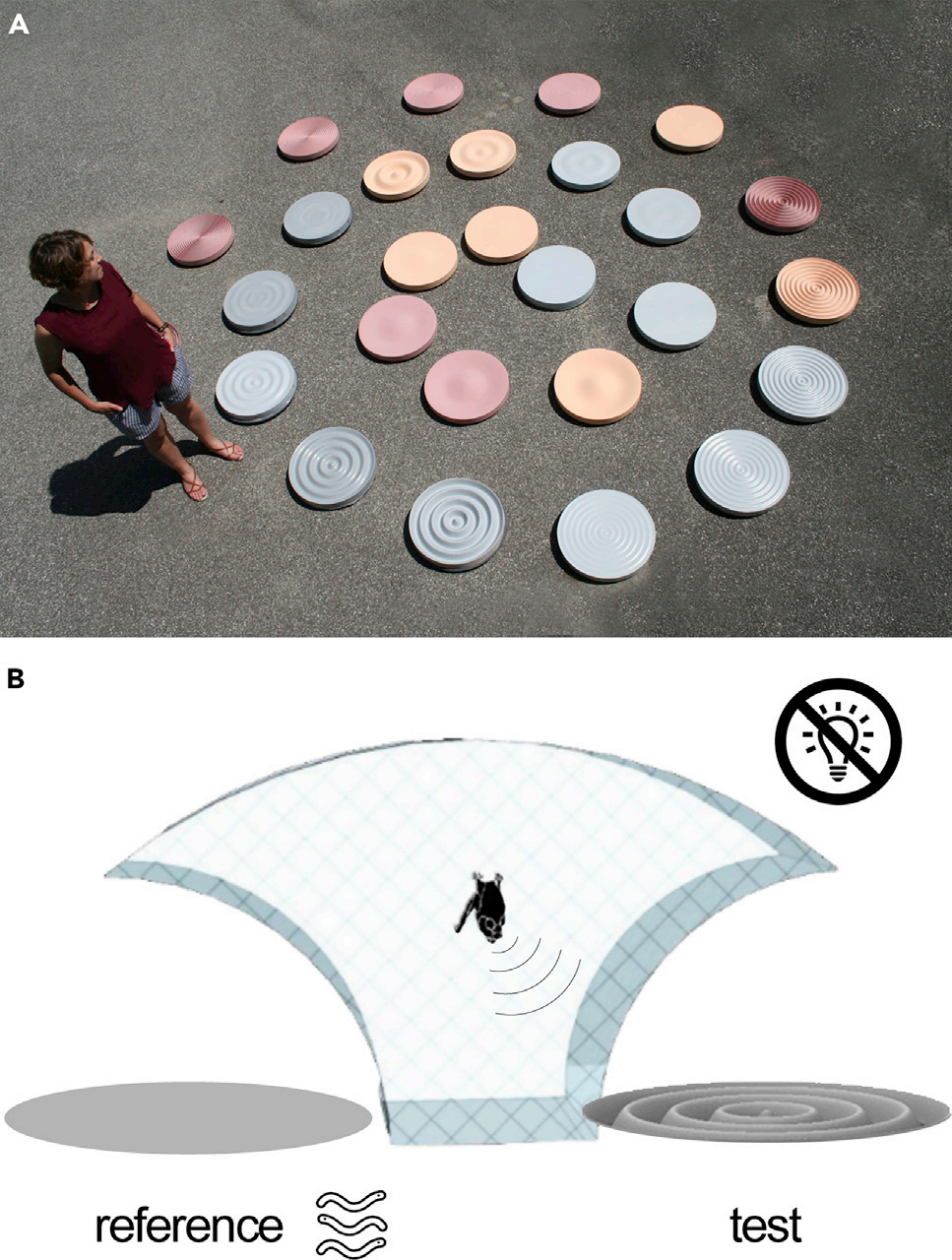
Experimental Stimuli and Setup (A) We simulated natural wave patterns with disks covered in concentric depth gratings. (B) Six bats discriminated in darkness between the flat reference disk and the test disk with wave patterns of varying wave amplitude and spatial frequency. To get a food reward (mealworms), bats indicated the pseudorandomly chosen position of the reference disk by crawling toward it from the starting position after echolocating toward both disks from inside a chicken-wire cage.

We generated 26 stimulus disks (45 cm diameter) with concentric wave patterns, simulating snapshots of water waves (Figure 1A). The wave patterns represented five different spatial frequencies (4.4 cyc/m, 8.9 cyc/m, 17.8 cyc/m, 35.6 cyc/m, and 71.1 cyc/m) at five different wave amplitudes (32 mm, 16 mm, 8 mm, 4 mm, 2 mm peak-to-peak), with one additional wave amplitude of 1 mm in the highest spatial frequency set. As control and reference, we used two completely flat disks (Figure 1B).

We measured the acoustic impulse response (IR) of each stimulus disk. The IR of an object describes the object's properties: it can be seen as the echo-acoustic picture of the object. As a visual image is a reflection pattern in response to a flash of white light, the IR describes the reflections of a 3D scene when it is ensonified with a spectrally white acoustic impulse. Convolving the IR with a bat's emitted call will recreate the echo as received by the bat. The target strength of an object is a measure of its reflective strength and is directly proportional to the echo level. We calculated target strength differences between each rippled test disk and the flat reference disk as a measurement of the relative echo level. We further investigated the temporal features of the IRs.

**Figure 2:**
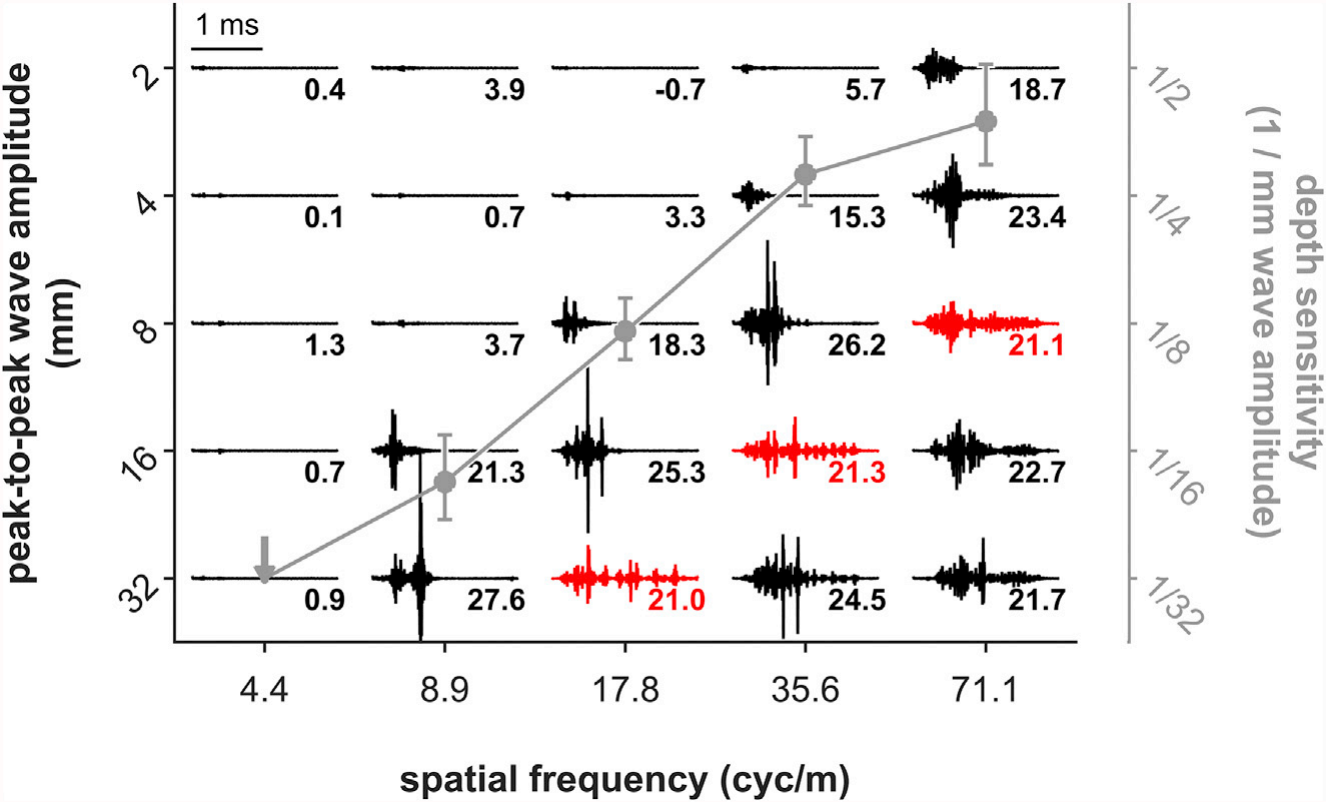
Measured Impulse Responses (IRs) of All Wave Patterns Amplitude and duration of IRs increase with increasing wave amplitude (top to bottom) and spatial frequency (left to right). Small numbers indicate target-strength difference (TSD) between each rippled disk and the reference disk. Depicted in red are exemplary IRs of different spatial frequency and wave amplitude but not target strength; these IRs differ in their temporal structure. Overlaid in gray is the mean (± SEM) sensitivity function as in Figure 4.

IRs (and therefore echoes) become louder and longer with increasing wave amplitude (Figure 2). This effect occurs at smaller wave amplitudes for higher spatial frequencies compared with lower spatial frequencies. Altogether, target strength difference increases for higher spatial frequencies and higher wave amplitudes.

**Figure 3:**
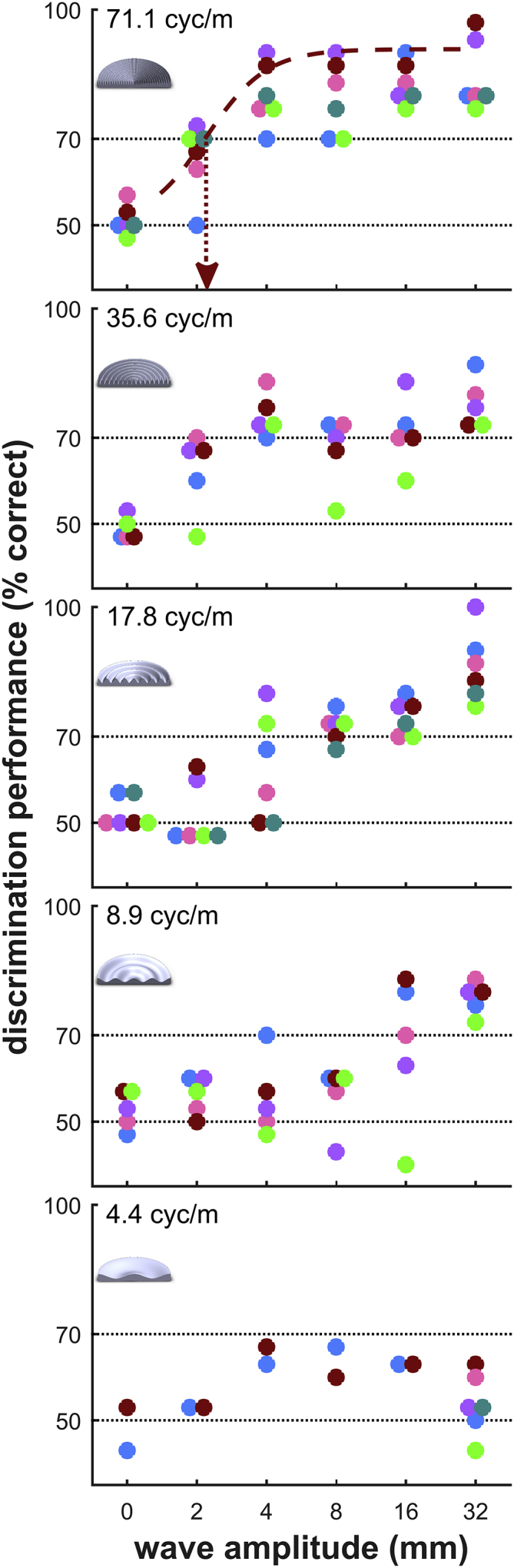
Wave-Pattern Discrimination Reveals Sensitivity to Patterns of High Spatial Frequencies in Echo-Imaging Wave-pattern discrimination performance of six bats (colored dots, n = 30 trials per dot) as a function of wave amplitude improves with increasing spatial frequency (bottom panel to top panel). Discrimination thresholds were extracted from the fitted psychometric functions at 70% correct (dashed line in top panel shows exemplary sigmoid fit, arrow points toward extracted threshold, which can be found again in Figure 4). Horizontal dotted lines at 50% and 70% correct depict chance and significance (p < 0.05) levels, respectively. Insets show exemplary patterned disks with 32-mm wave amplitude.

**Figure 4:**
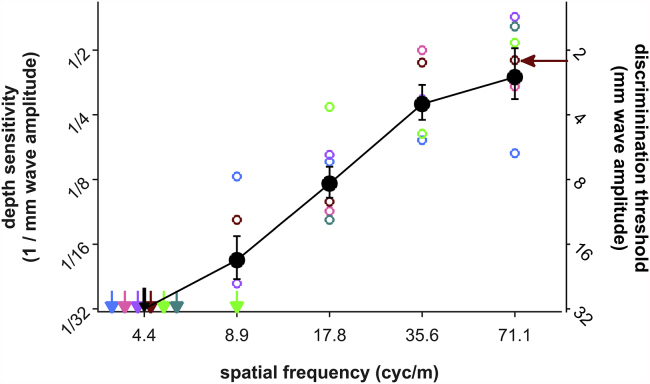
Sensitivity Function Individual (colored) and mean (black, ± SEM) depth sensitivity (the reciprocal of the detection threshold as extracted from Figure 3) improves with increasing spatial frequency. Where no threshold was found within the range of tested wave amplitudes (downward-pointing arrows), threshold was assumed to be higher than 32 mm, but set to 32 mm for calculations. The arrow in the top right corner points out the threshold value extracted from the exemplary sigmoid fit in the top panel of Figure 3.

Bats (*Phyllostomus discolor*) were trained in a two-alternative, forced-choice paradigm with food reward to choose between a flat reference disk and a disk with concentric waves of varying amplitude and spatial frequency (Figure 1B). After bats discriminated a large wave amplitude of 32 mm at a spatial frequency of 17.8 cyc/m to criterion (70% correct), we stepwise reduced wave amplitude, collecting 30 trials per wave amplitude. We then measured the psychometric functions for four more spatial frequencies in the same way. The experiments yielded one psychometric function per bat and spatial frequency, i.e., the discrimination performance as a function of wave amplitude at each spatial frequency (Figure 3). For all bats and across all spatial frequencies, discrimination performance was poor at low wave amplitudes and improved with higher wave amplitudes (Figure 3). For instance, at a spatial frequency of 17.8 cyc/m, wave amplitudes up to 2 mm were not discriminated from a flat disk above chance level. Discrimination improved as wave amplitude increased, reached threshold level at 8 mm in five of six bats, and improved further with larger wave amplitudes of 16 and 32 mm. Thus echo-imaging is more sensitive to high waves than to low waves. This pattern existed for all spatial frequencies, yet with frequency-dependent differences: whereas at the lowest presented spatial frequency (4.4 cyc/m) none of the bats detected even the highest wave amplitude (32 mm), the bats reliably detected 2-mm waves at the highest spatial frequency of 71.1 cyc/m. We estimated the actual discrimination thresholds for each spatial frequency and animal from a fitted sigmoidal function at 70% correct performance (p < 0.05, binomial test; cf. Figure 3, top panel, dashed red line). Combined, the extracted threshold values render the sensitivity function, with sensitivity being the reciprocal of the discrimination threshold (Figure 4). Sensitivity improved with increasing spatial frequency: all bats required higher waves to detect low spatial frequencies, whereas lower waves sufficed with increasing spatial frequencies.

## Discussion

The biophysical parameters of wave patterns parallel the behavioral detection thresholds: target-strength differences (TSD) increased with increasing wave amplitude and spatial frequency, with a very sudden increase around the wave-amplitude threshold (Figure 2). The disks' TSD at discrimination threshold for each spatial frequency roughly matches *Phyllostomus discolor*'s threshold for TSD of about 5–7 dB (Heinrich et al., 2011), suggesting that spatial frequency sensitivity is mediated by TSD. However, to discriminate spatial frequencies with equal target strengths, bats would have to abandon target-strength evaluation and most likely analyze temporal or spectral features of the echoes (compare colored IRs in Figure 2).

**Figure 5:**
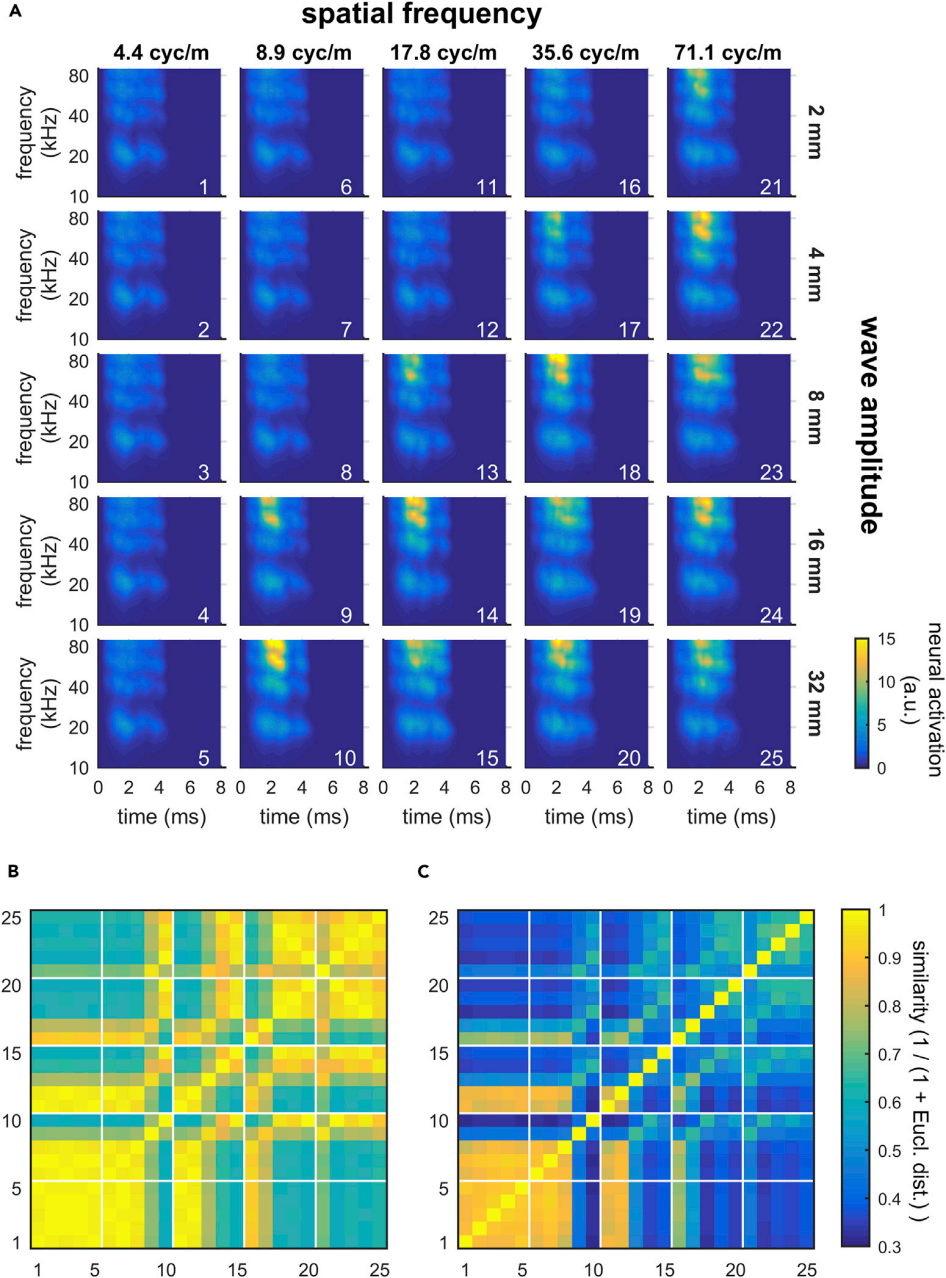
Neural Activation Patterns (NAPs) of Wave Patterns Represent the Information Available to the Bat Central Auditory System (A) Echoes from wave patterns are processed according to a physiologically plausible model of the auditory periphery (Wiegrebe, 2008). The resulting NAPs reflect the multi-harmonic structure of the bats' echolocation calls along the frequency axis and the time course of neural excitation in each frequency channel along the time axis. Wave patterns with lower wave amplitude (top rows) and lower spatial frequency (left columns) evoke weaker neural activation. White numerals from 1–25 number different wave patterns. (B) Confusion matrix for similarities between the mean activations (averaged across frequency and time) of the NAPs evoked by echoes from the different wave patterns. At low spatial frequencies (lower left corner), similarity is very high between different wave amplitudes. At high spatial frequencies (upper right corner), similarity is also high between different wave amplitudes. Similarity is calculated as the reciprocal of the Euclidean distance plus 1 and can range from 1 (equal) to 0 (fully dissimilar = infinite Euclidean distance). Axis labels refer to the white numerals in (A). (C) Confusion matrix for similarities between the activations of the NAPs evoked by echoes from the different wave patterns. Here similarity is calculated on the NAPs without averaging, exploiting the spectro-temporal distribution of activations in the auditory periphery. At low spatial frequencies (lower left corner), similarity is high between different wave amplitudes, but lower than the corresponding similarities in (B). Notably, at high spatial frequencies (upper right corner), similarity is only medium between different wave amplitudes and much lower than the corresponding similarities in (B). Overall, all similarity values are lower than their equivalents in (B). Again, similarity is calculated as the reciprocal of the Euclidean distance plus 1 and can range from 1 (equal) to 0 (fully dissimilar = infinite Euclidean distance). Axis labels refer to the white numerals in (A).

We addressed the perceptual features available to the bats for the discrimination of different wave patterns from the flat reference disk as well as from one another. We created echoes as they would be reflected off the wave patterns by convolving a typical echolocation call with a wave pattern's respective IR. We then recruited a physiologically plausible model of the bats' peripheral auditory processing (Wiegrebe, 2008) to calculate neural activation patterns (NAPs) (Patterson et al., 1995, Wiegrebe, 2008). NAPs are qualitatively similar to physical spectrograms, but importantly they possess physiologically plausible temporal and spectral resolution. NAPs represent the information available to the bat central auditory system. The NAPs that we created for the different wave patterns reflect the multiharmonic structure of the bats' echolocation calls along the frequency axis and the time course of neural excitation in each frequency channel along the time axis (Figure 5A). They further confirm our previous analysis (Figure 2) that echo amplitude increases for higher spatial frequencies and higher wave amplitudes.

We gained deeper insight into the echoes' information content from similarity analyses. We calculated similarities between echoes from each and every wave pattern as the reciprocal of the sum of the Euclidean distance and 1 (Segaran, 2007) for two echo parameters: as first parameter, we used the averaged activation of the different NAPs, i.e., we disregarded the spectro-temporal distribution of activation and only used its overall magnitude, a measure related to overall echo amplitude (confusion matrix in Figure 5B). As second parameter, we used the activation of the different NAPs without averaging across time and frequency, i.e., the analysis exploited the spectro-temporal distribution of activation in the bat's auditory periphery (confusion matrix in Figure 5C). The amplitude-only analysis (Figure 5B) indicates very high similarity between different wave amplitudes at low spatial frequencies and high similarity between different wave amplitudes at high spatial frequencies. When we compare this magnitude-based confusion matrix to that of the NAPs with the temporal and spectral information taken into account (Figure 5C), the similarities between different wave amplitudes at low spatial frequencies remain, but they are slightly reduced. Furthermore, the similarities between different wave amplitudes at high spatial frequencies are strongly reduced in the full-NAP-based confusion matrix. Generally, all similarity values based on NAPs with spectro-temporal information are lower than their equivalents based on overall magnitude alone. This observation suggests that the physiologically plausible NAPs contain ample information for the bats to discriminate between different wave patterns with high spatial frequencies even when these have similar overall amplitude—a hypothesis that remains to be evaluated with behavioral experiments.

**Figure 6:**
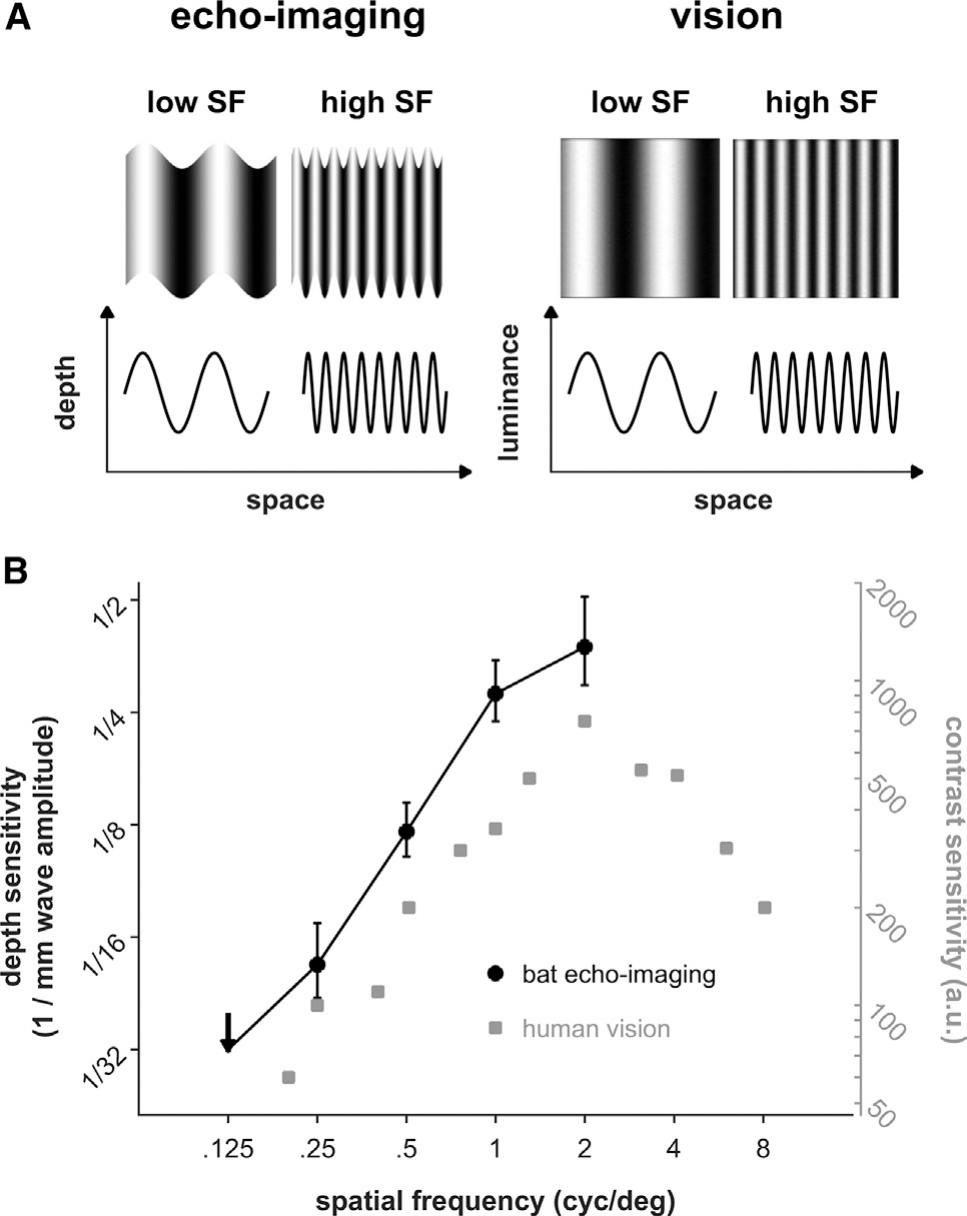
Spatial Frequency in Echo-Imaging and Vision (A) Spatial frequency (SF) quantifies the periodic change of either depth (echo-imaging) or luminance (vision) along a spatial axis. (B) The high-pass characteristic of the current depth sensitivity in bat echo-imaging (black circles; mean ± SEM) is well comparable to the initial high-pass part of the luminance-contrast sensitivity in human vision (gray squares: black-and-white contrast sensitivity, adapted with permission Campbell and Robson, 1968). Spatial frequency is expressed in units of cycles per degree observation angle. For the bat data, the unit conversion is based on the bats' typical viewpoint in the setup at 45° and 40 cm distance from the disk center.

Spatial frequency is a measure most commonly used in visual research. Since the pioneering work of Wiesel and Hubel on simple and complex receptive fields (Wiesel and Hubel, 1966), the concept of spatial frequency has proved to be one of the most powerful tools to understand visual perception. A basic stimulus for visual research is the sine wave grating, a pattern whose luminance profile varies sinusoidally over space (Figure 6A). Spatial frequency refers to the number of light and dark cycles per meter (cyc/m). The contrast needed by any visual system to perceive sine wave gratings changes as a function of spatial frequency. The contrast sensitivity function describes this relationship (Campbell and Robson, 1968, Enroth-Cugell and Robson, 1966). As the required contrast also depends on viewing distance, spatial frequency is expressed as cycles per degree of visual angle (cyc/deg). High spatial frequencies, i.e., steep changes of luminance along one spatial axis, represent local object features (e.g., object edges), whereas low spatial frequencies code for more global information about the object's shape (Bar, 2004). In images that contain both low and high spatial frequencies at similar proportions, the high frequencies are perceived as the figure and the low frequencies as the background. The larger the spatial frequency difference, the more pronounced is the perceptual figure-ground separation (Klymenko and Weisstein, 1986).

The wave patterns used in this study represent an echo-acoustic equivalent of a sine wave grating: a surface that changes its depth periodically instead of its luminance (Figure 6A). Therefore we can express the spatial frequency of the wave patterns in units of cycles/degree observation angle and compare sensitivity in echo-imaging and vision (Figure 6B). For spatial frequencies up to 2 cyc/deg, the human sensitivity function for visual contrast shows high-pass properties. This is qualitatively comparable to the high-pass shape of the sensitivity function we measured in *P*. *discolor*. As luminance and depth sensitivity cannot be compared directly, the y-axis in Figure 6B is arbitrarily scaled. Also, note that human visual contrast sensitivity drops off at spatial frequencies above 2 cyc/deg, resulting in an overall band-pass characteristic of the visual contrast sensitivity function. Here we could not investigate spatial frequencies above 2 cyc/deg owing to manufacturing limitations. The question whether echo-imaging has the same overall band-pass characteristic as vision remains open. What we can conclude is that both sensitivity functions show a high-pass characteristic between 0.125 and 2 deg/cyc. We speculate that this allows both vision and echo-imaging to segregate high-frequency objects of interest (”figure”) from low-frequency background (“ground”), that is, to extract contours of objects in a complex scene.

Many bat species from at least three families exploit the abundance of prey—often soft-bodied and easily digestible—offered by water bodies (Denzinger and Schnitzler, 2013, Fukui et al., 2006). Although several studies point toward an impairment of foraging efficiency through clutter echoes or noise produced by water turbulences (Boonman et al., 1998, Mackey and Barclay, 1989, Rydell et al., 1999, Siemers et al., 2001, Von Frenckell and Barclay, 1987, Warren et al., 2000), a more recent study indicates that neither prey detection nor discrimination are impaired by turbulent water: *Myotis daubentonii* perform equally over rippled, flowing water in the field and over still water in the laboratory (Zsebők et al., 2013). This discrepancy suggests that the degree and extent of turbulence (and thus the spatial frequency composition) determines the degree of prey detection impairment. We speculate that the bat auditory system exploits an environmental high-pass filter to assess spatial frequency and segregate foreground from background. We propose the existence of a threshold spatial frequency below which turbulent water surfaces do not impair prey detection and above which turbulent water surfaces impair prey detection and are therefore avoided by foraging bats.

Rydell et al. (1999) report reduced activity of *M. daubentonii* over water with ripples that were approximately 5–10 cm in wavelength and 2–3 cm in amplitude. This corresponds to spatial frequencies of 10–20 cyc/m with magnitudes of 20–30 mm. Siemers et al. (2001) found drastically reduced capture performance over a clutter screen that introduced spatial frequencies of about 69 cyc/m with a magnitude of 2.5 mm. Here we have reported detection thresholds of around 8 mm for a spatial frequency of 17.8 cyc/m and thresholds of around 1.5 mm for a spatial frequency of 71.1 cyc/m. It follows that both the ripples that Rydell et al. (1999) observed and the clutter that Siemers et al. (2001) presented would have been noticeable to the bats and indeed have affected prey detection. Zsebők et al. (2013) found reduced detection performance over a clutter surface of artificial grass, consisting mainly of sharp edges. The duckweed vegetation of Boonman et al. (1998) included sharp edges as well. Again we conclude that high spatial frequencies, as introduced by sharp edges, hinder foraging bats.

In contrast, *M*. *daubentonii* performed similarly above smooth water in the laboratory and above rippled water in the field, i.e., echoes off rippled water did not impair the bats' performance (Zsebők et al., 2013). On large water bodies, wind generates waves that typically have rather high amplitudes (0.8–38.3 mm) but long wavelengths of around 82 cm (Bleckmann and Rovner, 1984), corresponding to a spatial frequency of less than 1.5 cyc/m. Given that in our study none of the bats detected wave patterns of 4.4 cyc/m spatial frequency at a wave amplitude of 32 mm or lower, we believe that bats are highly unlikely to perceive wind-generated waves. We conclude that in the perception of an echolocating bat, faintly turbulent water surfaces equal smooth water surfaces and do not impair prey detection.

In summary, our work offers insights into the common ground between visual and auditory system. We have obtained compelling evidence that the physical properties of waves provide the auditory system with a perceptive cue directly related to spatial frequency. The functional similarities yet mechanistic differences between visual and auditory system signify convergent evolution of spatial-information processing.

### Limitations of the Study

Our study species *P. discolor* is not a trawling bat. However, it uses short and broad-band echolocation calls like the European trawling bat *M. daubentonii* (Kalko and Schnitzler, 1989, Rother and Schmidt, 1982). Thus, both species are equipped with comparable sonar qualifications for the detection task. We assume that a water specialist like *M*. *daubentonii* would especially benefit from spatial-information processing in echo-imaging and thus might even be more sensitive to spatial frequency differences.

## Methods

All methods can be found in the accompanying Transparent Methods supplemental file.

## Data and Software Availability

The data of this paper is available at Mendeley Data (https://doi.org/10.17632/86vcbkxdht.2).

## Supplemental Information

Document S1. Transparent Methods
