# Supplementary material for: Echo-Imaging Exploits an Environmental High-Pass Filter to Access Spatial Information with a Non-Spatial Sensor
Source: iScience. 2019 Apr 18;14:335–44. doi: 10.1016/j.isci.2019.03.029 (PMC6491417; doi:10.1016/j.isci.2019.03.029)
Supplement: Document S1. Transparent Methods [file mmc1.pdf]

ISCI, Volume *14*

Supplemental Information

Echo-Imaging Exploits an Environmental High-Pass Filter to Access Spatial Information with a Non-Spatial Sensor

A. Leonie Baier, Lutz Wiegrebe, and Holger R. Goerlitz

Transparent Methods

Stimuli

The 28 stimulus disks (Fig. 1A) were manufactured by a milling cutter (Modellbau Grossmann, Calw). They had a height of 5 cm, a diameter of 45 cm and were covered in concentric waves. At the typical distance (40 cm) and ensonification angle (45°), the disks covered a considerable area of the bat's sonar footprint (Vanderelst et al., 2010). The cross-section of each disc's surface can be described by a sine wave function whose peak amplitude represents the wave amplitude and whose number of periods per diameter defines the spatial frequency. Five spatial frequencies were tested, namely 4.4 cyc/m, 8.9 cyc/m, 17.8 cyc/m, 35.6 cyc/m, and 71.1 cyc/m, corresponding to 2, 4, 8, 16 and 32 wave troughs per disc, respectively. From the bat's typical viewpoint in the setup (40 cm decision distance and 45° ensonification angle), this corresponds to spatial frequencies of about 0.125 to 2 cycles per degree observation angle (cyc/deg). Within each spatial frequency, a set of six wave amplitudes was tested; namely 32 mm, 16 mm, 8 mm, 4 mm, 2 mm, and 0 mm peak-to-peak amplitude. One additional disk with 1 mm wave amplitude was created for the 71.1 cyc/m set. The reference stimulus was a flat disc, identical to the 0 mm control.

Impulse Responses and Neural Activation Patterns

For evaluation of the echo scenes reflected back by the experimental disks, we ensonified them with band-pass filtered white noise through a loudspeaker (Vifa Denmark A/S, Viborg, Denmark) and amplifier (AVR 445, Harman/Kardon, Stamford,

CT, USA). We recorded the echoes with a ¼'' measurement microphone and pre-amplifier (Type 40BF and 26CA, G.R.A.S. Sound & Vibration A/S, Holte, Denmark; protective grid removed) powered by a power module (G.R.A.S. Type 12AA). We obtained impulse responses through cross-correlation in Matlab (Version R2015a, The MathWorks Inc., Natick, MA, USA). Target strength differences between rippled and smooth disks were calculated by subtracting the root mean square of the corresponding impulse responses.

Sound recording and noise playback were synchronized through an audio interface (Fireface 800, RME Audio AG, Haimhausen, Germany) which was controlled by SoundMexPro software (HörTech, Oldenburg, Germany) in Matlab. The noise pass band ranged from 3 to 92 kHz. This encompasses the main frequency range used by *P. discolor*. Impulse response measurements were carried out with the speaker and microphone positioned at a distance of 40 cm to the disk at angles of 30°, 45°, 60° and 90° relative to the disc's centre. The typical position of a bat during the detection task was at 40 cm distance and 45°. We ensured a flat frequency response of the noise playback by filtering white noise with the speaker's compensatory impulse response (impulse response filter with cut-off frequencies of 3 and 92 kHz). Initial measurements of the speaker impulse response were conducted with the measurement microphone oriented perpendicular to the speaker at a distance of 40 cm.

To address the perceptual features by which the bats may discriminate the wave patterns with different spatial frequencies and wave amplitudes from the flat reference disk (and potentially from one another), we recruited a physiologically plausible model of the bats' peripheral auditory processing. Specifically, we convolved the measured

impulse responses of the different wave patterns with a typical *P. discolor* echolocation call to create an echo as it would be received by the bat. This echo undergoes realistic inner-ear processing with a bank of constant-Q auditory filters with physiologically plausible, frequency-dependent bandwidths (Wiegrebe, 2008). Inner ear transduction is simulated by half-wave rectification followed by exponential amplitude compression and subsequent low-pass filtering to simulate the sluggishness of the inner hair cell receptor potential. This results in a neural activation pattern (NAP) (Patterson et al., 1995; Wiegrebe, 2008), which is qualitatively similar to a physical spectrogram but with physiologically plausible temporal and spectral resolution.

For similarity analyses, we calculated similarities between the echoes from each and every wave pattern as the reciprocal of the sum of the Euclidean distance and 1 (Segaran, 2007). We calculated the Euclidean distance (and thus similarity) for two different echo parameters: first, we used the average activation of the different NAPs, calculating the Euclidean distance as the square root of the squared difference between one NAP's mean across both time and frequency, and another NAP's mean across both time and frequency. Second, we calculated Euclidean distances for the different NAPs as the square root of the mean across both time and frequency of the squared difference between one NAP and another NAP. By averaging across time and frequency before and after subtracting signals, respectively, the spectro-temporal distribution of activations that was present in the NAPs was disregarded in the first simulation, but fully exploited in the second simulation.

Experimental Animals

We conducted experiments with six individuals (four male, two female) of the neotropical omnivorous species *Phyllostomus discolor* (Nowak, 1994). It emits short (<3 ms), downward frequency-modulated, multi-harmonic echolocation calls covering the frequency range between 45 and 100 kHz (Rother and Schmidt, 1982). They originated from a breeding colony in the Department Biology II of the Ludwig Maximilians University in Munich and were kept at the Max Planck Institute for Ornithology in Seewiesen (12 h night / 12 h day cycle, 65-75 % relative humidity, 28 °C). In the housing room, bats had unlimited access to water at all times and to mixed fruit and mealworms (larvae of *Tenebrio molitor*) supplemented with minerals (Korvimin ZVT+Reptil®), vitamins (NutriCal®), and essential fatty acids (Efaderm®) during non-training days. During training days, bats were fed in the experiment. The experiment complied with the principles of laboratory animal care and was conducted under the regulations of the current version of the German Law on Animal Protection (approval 55.2-1-54-2532-34-2015, Regierung von Oberbayern).

Experimental Setup

The experiments were performed inside a dark echo-attenuated chamber in a dark echo-attenuated room. Two infrared floodlights (TV6819, ABUS, Wetter, Germany) and a camera (WAT-902H2 Ultimate, Watec Co. LTD, Higashine, Japan) were mounted inside the chamber for observation. Both experimenter and control computer were stationed outside the chamber. Inside the chamber, a custom-built table held both the stimulus-presentation apparatus and the experimental cage (87 cm x 65 cm x 18 cm, W

x H x D, built from wire mesh; Fig 1B). Two stimulus disks, reference plus test, were presented simultaneously. Both disks were placed on a carousel mechanism of the stimulus-presentation apparatus underneath the table, swivelled to their assigned positions and hoisted up into two holes in the tabletop (Fig 1B). Due to this procedure, blinding was not possible. Light barriers were fixed close to the bottom of both cage sidewalls to monitor the presence of the bat. They were connected to the serial port of a personal computer. Data recording and stimuli presentation order were controlled via a custom Matlab R2007b application (The Mathworks, Inc., Natick, MA, USA).

General Procedure

Daily training sessions lasted 20-30 minutes per bat at 5 d per week, followed by a 2 d break. The experiment followed a two-alternative, forced-choice paradigm (2AFC) with food reinforcement. Once a bat sat at the starting position in the cage, both the smooth disk (reference) and one rippled disk (test) were presented. The position of the smooth disk (left or right) was pseudorandom (Gellermann, 1933). Bats had to move towards the smooth disc, where they were rewarded with a mealworm as soon as they interrupted the corresponding light barrier. Then the bats returned to the starting position, the disks were swivelled and a new trial started. Once a bat had learned this task (>70% correct choices on five consecutive days), data acquisition started for the same spatial frequency but with stepwise smaller wave amplitudes, making the detection task more and more difficult. Daily data acquisition started with four consecutive trials presenting the largest wave amplitude of 32 mm, continued with stepwise-reduced wave amplitudes, each presented for four trials. After a wave

amplitude of 0 mm was reached, data acquisition returned to 32 mm etc. until the daily session was completed. To keep the bats motivated, three or two trials instead of four trials per wave amplitude could be presented in a row or easier trials (with larger wave amplitude) could be interspersed. Testing of one spatial frequency set was completed when 30 trials per wave amplitude and bat were recorded.

For the lowest tested spatial frequency of 4.4 cyc/m, none of the bats reached the criterion level of 70% correct choices. To keep up motivation and to exclude the possibility that the bats had unlearned the task, trials with disks from a higher spatial frequency set were interspersed. Like this, testing of all wave amplitudes at the 4.4 cyc/m spatial frequency could be completed for two bats. For the other four bats, we needed to adjust the procedure further. We tested the detection of the spatial frequency of 4.4 cyc/m only for the largest wave amplitude of 32 mm and interspersed trials with the next higher spatial frequency of 8.9 cyc/m at the largest wave amplitude of 32 mm (which the bats could detect). In this manner, we controlled for the possibility that lack of motivation or forgetting the task caused the bats to miss the criterion level at a spatial frequency of 4.4 cyc/m. After none of the bats reached the criterion level at the largest wave amplitude at a spatial frequency of 4.4 cyc/m, testing of the remaining smaller wave amplitudes was dismissed. We conclude that bats cannot perceive ripples of 32 mm wave amplitude or smaller at a spatial frequency of 4.4 cyc/m. Bat 6 dropped out due to pregnancy before completion of the 8.9 cyc/m and 35.6 cyc/m data sets.

Statistical Analysis

Psychometric functions for five spatial frequencies between 4.4 cyc/m and 71.1 cyc/m were measured, i.e. the detection performance of the bat (in percentage correct detections over 30 trials) as a function of wave amplitude (from 32 mm to 0 mm). A sigmoid function was fitted to the psychometric function (Matlab R2007b; The Mathworks, Inc., Natick, MA, USA) and the value of this fit at 70% was taken as threshold (corresponding to $p = 0.02$ in a binomial test with 30 trials). For the spatial frequency 4.4 cyc/m, the threshold was assumed higher than 32 mm, as it was for bat 5 at the spatial frequency 8.9 cyc/m where it missed the 70% criterion. For calculations of the average performance across bats, the threshold value was conservatively set to 32 mm.

Data and Software Availability

The data of this paper is available at Mendeley Data (doi.org/10.17632/86vcbkxdht.2).

Supplemental References

Gellermann, L.W. (1933). Chance orders of alternating stimuli in visual discrimination experiments. Ped. Sem. J. Gen. Psychol. 42, 206-208.

Nowak, R.M. (1994). Walker's bats of the world (JHU Press).

Vanderelst, D., De Mey, F., Peremans, H., Geipel, I., Kalko, E., and Firzlaff, U. (2010). What noseleaves do for FM bats depends on their degree of sensorial specialization. PLoS One 5, e11893.

Patterson, R.D., Allerhand, M.H., and Giguere, C. (1995). Time-domain modeling of peripheral auditory processing: A modular architecture and a software platform. J. Acoust. Soc. Am. 98, 1890-1894.

Rother, G., and Schmidt, U. (1982). The influence of visual information on echolocation in Phyllostomus discolor (Chiroptera). Z. Säugetierk. 47, 324-334.

Wiegrebe, L. (2008). An autocorrelation model of bat sonar. Biol. Cybern. 98, 587-595.
